# A Randomized, Controlled Trial of the Novel, Potent Kv7 Channel Opener Azetukalner in Individuals with Major Depressive Disorder and Anhedonia: Neural Response to Reward, Clinical Outcomes, and Safety

**DOI:** 10.21203/rs.3.rs-7448148/v1

**Published:** 2025-09-15

**Authors:** James Murrough, Rachel Fremont, Philipp Neukam, Usha Govindarajulu, Jessica Ables, Sara Hameed, Marcella Corwin, Mackenzie Hargrove, Helena Chang, Sarah Boukezzi, Chris Kelly, Alan Swann, Ramiro Salas, Dania Amarneh, Julia Engelhardt, Andreas Weyland, Emilia Bagiella, Laurel Morris, Sanjay Mathew

**Affiliations:** Icahn School of Medicine at Mount Sinai; Icahn School of Medicine at Mount Sinai; Icahn School of Medicine at Mount Sinai; Menninger Department of Psychiatry and Behavioral Sciences, Baylor College of Medicine, Houston TX, USA; Depression and Anxiety Center for Discovery and Treatment, Department of Psychiatry, Icahn School of Medicine at Mount Sinai; BCM

## Abstract

Major Depressive Disorder (MDD) is a common and debilitating disease. Kv7 potassium channels are relevant to reward processing and represent a novel target for depression and anhedonia. Azetukalner is a positive allosteric modulator of Kv7. This phase II, randomized, double-blind, placebo-controlled trial assessed changes in brain reward function, clinical outcomes, and safety following treatment with azetukalner or placebo in individuals with MDD and anhedonia. The study included 60 participants with MDD and anhedonia in a current major depressive episode. Participants were randomized 1:1 to receive azetukalner (20 mg orally once daily with food) or placebo for 8 weeks. The primary endpoint was change in bilateral ventral striatum (VS) activity assessed by fMRI during a reward task from baseline to week 8. Secondary endpoints included changes in depression severity and anhedonia measured by the Montgomery-Åsberg Depression Rating Scale (MADRS) and Snaith-Hamilton Pleasure Scale (SHAPS). Of 60 participants, 29 were randomized to azetukalner and 31 to placebo. There was no significant difference in VS response to reward anticipation between groups. Compared to placebo, azetukalner was associated with numerical benefit on the MADRS and SHAPS that did not reach statistical significance. Additional exploratory endpoints also favored azetukalner over placebo. Discontinuation rates due to adverse events were low and did not differ across groups. Despite not meeting the primary neuroimaging endpoint,secondary and exploratory outcomes suggested potential improvement in depressive and anhedonia symptoms, consistent with recent studies.

**Trial Registration:**

Clinicaltrials.gov ID NCT04827901

## INTRODUCTION

Major Depressive Disorder (MDD) is one of the most disabling medical conditions worldwide and current available treatments inadequately address this large public health burden. Anhedonia – the reduced ability to experience or anticipate pleasure – is a core feature of depression and greater severity of anhedonia is associated with a chronic disease course, increased suicide risk, and poor response to treatments (1). Anhedonia is linked to dysfunction within the brain reward system, including mesocorticolimbic circuits (2, 3) that encompass the ventral tegmental area (VTA) and the ventral striatum (VS) (2–4).

Basic work utilizing rodent chronic social stress models indicate that animals displaying susceptibility to stress by evidencing pro-depressive and anhedonic-like behavioral changes show increased phase firing rate within dopaminergic neurons of the VTA projecting to VS (5, 6). Critically, animals resilient to stress that do not develop pro-depressive behaviors show up-regulation of voltage-gated potassium (K+) channel functions within the VTA compared to susceptible and stress-naïve mice (7, 8). In particular, resilient mice display up-regulation of Kv7 (a.k.a., KCNQ)-type K + channels within VTA neurons that control neuronal excitability and phasic firing within the VTA-VS circuit. Local infusion within the VTA or peripheral injection of positive allosteric openers of Kv7 channels show antidepressant behavioral signals in rodent stress models (9–11).

Translating this work to humans, our group previously tested the neural and behavioral effects of the first-in-human Kv7 opener ezogabine (a.k.a., retigabine; approved as an anti-seizure drug in the US) in adults with MDD (12, 13). In an open-label clinical trial treatment with up to 900 mg ezogabine total daily for 5 weeks was associated with improvements in depression severity and anhedonia as measured by the Montgomery-Åsberg Depression Rating Scale (MADRS) and Snaith-Hamilton Pleasure Scale (SHAPS), respectively, and increased behavioral sensitivity to reward, measured using a computerized objective measure of reward leaning (13). In a second, randomized, placebo-controlled trial (RCT), adults with MDD and elevated levels of anhedonia were randomized to ezogabine up to 900 mg daily for 5 weeks or matching placebo (REF). Change in VS activity during reward anticipation measured using functional MRI and a variant of the monetary incentive delay task termed the incentive flanker task (IFT) represented the primary outcome. In that study, ezogabine led to a non-significant increase in VS response to reward compared to placebo in addition to significant improvements in depression and anhedonia.

Herein, we report the results of a phase II, proof-of-concept RCT comparing azetukalner, a novel, potent Kv7 opener to placebo in adults with MDD and anhedonia as monotherapy. In line with our previous RCT, response to reward anticipation within the VS represented the primary outcome.

## PATIENTS and METHODS

### Study Participants and Design

This parallel-arm, randomized, double-blind, placebo-controlled, multicenter clinical trial was conducted at the Icahn School of Medicine at Mount Sinai in New York City, NY, and Baylor College of Medicine in Houston, TX. Approval for the study was granted by both institutional review boards. The study was funded by the National Institutes of Health (NIH) and a NIH data and safety monitoring board oversaw trial progress and participant safety. Before any procedures were conducted, the research team secured written informed consent from all participants. Recruitment for the study was facilitated through advertisements and referrals from healthcare providers between October 2021 and August 2024.

The trial enrolled 60 participants (30 per site) between the ages of 18 and 65, with a primary diagnosis of MDD, as defined by the Diagnostic and Statistical Manual of Mental Disorders, Fifth Edition (DSM-5). The primary diagnosis was determined by a trained assessor using the Structured Clinical Interview for DSM-5 Research Version (SCID-5) (14). Participants also must have had clinically significant anhedonia (Snaith-Hamilton Pleasure Scale (SHAPS) ≥ 20) (15) and at least moderate illness severity (Clinical Global Impressions severity scale (CGI-S) ≥ 4) (16) at screening. Exclusionary diagnoses included any primary psychiatric diagnosis other than MDD as defined by DSM-5 or substance use disorder in the past 6 months, with the exception of nicotine use disorder. Participants were required to be off antidepressants and other prohibited medications with CNS activity for a duration equivalent to 5 half-lives of the medication at the time of randomization.

Evaluations were conducted at: screening, baseline (week 0), weeks 1, 2, 4, 6, 8 (primary outcome), and study exit (weeks 12–13). During screening (−6 weeks to −1 day before baseline), the study team confirmed participant eligibility. At baseline, eligible participants were enrolled and randomly assigned 1:1 to active treatment with azetukalner or placebo using a computer-generated randomization scheme developed by the data coordinating center (Mount Sinai). Randomization assignment was stratified by clinical center and a random permuted block design of sizes 2 and 4 was employed within each stratum. Participants, clinical assessors, and treating physicians were blinded to the randomization assignment. Following randomization, participants were instructed to take two 10 mg capsules of study drug once daily in the evening with food for a total daily dose of 20 mg for 8 weeks. Participants who did not tolerate 20 mg daily had the option to reduce their dose to 10 mg daily. Medication adherence was assessed using pill count and the study team monitored an online self-reported medication tracking log. At baseline and primary outcome visits, participants underwent an MRI scan during which they completed the incentive flanker task (IFT) (17–19). At each post-baseline study visit, participants completed self-reports, clinical rating scales administered by trained raters, routine clinical laboratory testing, and met with a study psychiatrist to evaluate adverse events, suicidality, and changes in medications. Participants were followed for 4 weeks after the primary outcome visit for safety.

### Endpoints

The primary endpoint was change in blood oxygenation level-dependent (BOLD) signal (i.e., ‘activation’) within the bilateral VS from baseline to week 8 as measured by fMRI during IFT (19). Secondary endpoints included changes in clinical measures of depression and anhedonia across baseline, and weeks 2, 4, 6, and 8. Severity of depression was assessed by a trained rater using the MADRS, a 10-item scale that is sensitive to overall change in treatment response and discriminates between response and non-response (20). Symptoms of anhedonia were measured using the SHAPS self-report which contains 14 items designed to assess the four primary hedonic domains (15).

Exploratory endpoints included self-assessment of depressive symptom severity using the Quick Inventory of Depressive Symptomatology-Self Report (QIDS-SR) and self-report measurement of anhedonia with the Temporal Experience of Pleasure Scale (TEPS) at baseline and weeks 2, 4, 6, and 8 (21). The TEPS produces two sub-scores of anticipatory pleasure (TEPS-AP) and consummatory pleasure (TEP-CP) (21). A study clinician also assessed participant global illness improvement and severity utilizing the Clinical Global Impressions improvement scale (CGI-I) and CGI-S rating scales. Adverse events (AEs) were reviewed by a study psychiatrist at each study visit through week 12 with pre-defined adverse events of special interest (AESI) focused on disturbances in thinking and perception, bladder and urethral symptoms, cardiovascular changes and ophthalmologic changes. See Supplemental methods for more information. Serious AEs (SAEs) were defined as a negative medical occurrence that resulting in death, life-threatening situations, hospitalization, disability, or birth defects.

### Incentive Flanker Task

The IFT task was completed as outlined by Costi and colleagues (12). We targeted the reward anticipation response in the VS by showing monetary cues indicating reward, loss or neutral, followed by a five-letter string. Participants responded to the center letter by pressing a response button with either their right index or middle finger. The response period duration varied by participant and was based on a titration process during a practice session. Participants were immediately presented with outcome feedback on trial performance (“Correct” or “Incorrect”). Participants completed four, 6-minute runs for a total of 120 pseudorandom trials, which were equally divided for each monetary cue (gain, loss, neutral). See Supplemental Methods for more information.

### Imaging Acquisition and Processing

MRI data were collected in a Siemens 3T Magnetom Skyra MRI scanner at the Icahn School of Medicine at Mount Sinai and in a Siemens 3T Prisma MRI scanner at Baylor College of Medicine; both sites used 32-channel head coils. A session included an anatomical, as well as functional scans for the IFT. The anatomical scan was acquired using a dual-inversion magnetization prepared gradient echo (MP2RAGE; 1×1×1 mm voxel resolution) and the functional images were acquired with TR = 1500 ms and 3×3×3 mm voxel resolution. All IFT fMRI data were preprocessed using multi-echo independent component analysis (ME-ICA), which includes slice-time correction, motion and physiological artifact removal. IFT subject level models were created with the main contrast of interest reflecting reward anticipation computed by subtracting the estimated parameters for the neutral condition from the parameters of the gain condition. The resulting contrast estimates were then extracted from the VS region of interest using an anatomical mask from the Harvard-Oxford atlas. The extracted data was then statistically analyzed (See **Supplemental Methods** for more details).

### Statistical Analysis

Sample size calculations were based on estimates from the ezogabine trial (12). Using a linear mixed-effects (LME) model with random intercept, a total of 50 participants (25 per group) would provide the trial with at least 80% power to detect a difference of 0.4 to 0.6 in bilateral VS activation from baseline to week 8 between azetukalner and placebo at a two-sided significance level of 0.10. To account for study dropout, the sample size was increased to 60 participants (See **Supplementary Methods** for more details).

Descriptive statistics were conducted on all outcomes with means and standard deviations calculated for continuous variables and counts and percentages for categorical variables. Efficacy analyses were performed in the intention-to-treat population which included all randomized participants identified by their initial group assignment. The primary endpoint was analyzed using an LME model and included treatment, week, and treatment-by-week interaction as fixed effects and a subject-specific intercept as a random effect. Participants with missing primary endpoint data had their bilateral VS activation value imputed using multiple imputation, under the assumption that the data were missing at random. The imputation model included randomization assignment and bilateral VS activation at baseline and week 8. The imputation process was repeated 30 times and analyses from these completed-and-imputed data sets were combined using Rubin’s rule (22) to test for differences between azetukalner and placebo through the treatment-by-week interaction term. The same modeling used for the primary outcome was employed for the analysis of the secondary and exploratory endpoints, using all available observations at baseline and weeks 2, 4, 6, 8, without imputation for missing outcomes. Least squares means were obtained to summarize within-group changes and treatment differences. Response to treatment was calculated as the percentage of participants with a reduction of at least 50% in MADRS score from baseline to week 8. Remission rate was computed as the percentage of participants who achieved a score of less than 10 on the MADRS at week 8. Participants with missing values on the MADRS at week 8 were considered failures for response and remission.

Safety analyses were performed in all randomized participants who received at least one dose of azetukalner or placebo. AEs were collected using MedDRA system organ classes and preferred terms over 12 weeks and summarized as incidence counts and percentages.

All analyses were performed at the two-sided 0.10 significance level using SAS version 9.4 (SAS Institute Inc.). Adjustment of the type I error rate for multiple testing across the secondary endpoints was not performed and no formal hypothesis testing was conducted on the exploratory endpoints as these were hypothesis-generating. All reported outcomes and analyses were prespecified.

## RESULTS

### Sample Characteristics:

A total of 89 consented participants were screened and 60 underwent randomization (**Supplemental Material, Figure S1**). The intention-to-treat population consisted of 29 participants in the azetukalner group and 31 participants in the placebo group. With 59 participants receiving at least one dose of study drug or placebo, the safety population for adverse events comprised 29 participants in the azetukalner group and 30 in the placebo group. One participant in the placebo group was lost to follow-up immediately after randomization and was excluded from the safety population. A total of 51 participants (85%; 24 [82.8%] in azetukalner and 27 [87.1%] in placebo) completed the 8-week treatment period. The demographic and baseline clinical characteristics are shown in Table 1. The mean (± SD) age was 38.1 ± 12.7 years, 40 (66.7%) were female, and 28/57 (49.1%) were White. The mean age of major depression onset was 21.5 years with 78% (39/50) having recurrent episodes.

### Primary Outcome

Of the 60 participants randomized, all had a pre-treatment fMRI scan and 53 (88.3%) completed the post-treatment fMRI scan. IFT task fMRI data were available for 53 participants with valid pre- and post-treatment scans, 57 participants had valid pre-treatment scans, two participants only had a valid post-treatment scan and for one participant no IFT data was available. At baseline, the study groups did not differ in their performance on the IFT as assessed by performance accuracy (azetukalner, 74.8%, SD = 3.5; placebo, 76.4%, SD = 3.3, p = 0.78).

**Figure 1:**
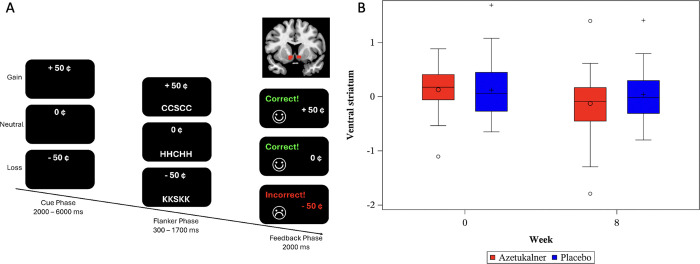
Activity Levels of the Ventral Striatum over the 8-Week Treatment Period A. Schematic representation of the Incentive Flanker Task (IFT) and coronal image of ventral striatum region of interest. B. Boxplots depict activity levels of the ventral striatum in response to reward anticipation during the IFT at baseline and week 8 in both groups. Higher levels indicate increased ventral striatum activity. Lines represent the median and circle/cross symbols are the arithmetic mean. The bottom of the box is the 25% percentile (lower quartile) and extends to the 75% percentile (upper quartile) at the top. The lower whisker is defined as 1.5*IQR below the 25th percentile. The upper whisker is defined as 1.5*IQR above the 75th percentile.

Activity levels in the bilateral VS during fMRI using IFT at baseline and week 8 is shown in Fig. 1 and reported in Table S2. Change in activation of the bilateral VS in response to reward anticipation was not significantly different between groups (difference in mean change from baseline at week 8, −0.16; 90% CI, −0.51 to 0.18; t = −0.79, p = 0.44).

### Secondary Outcomes – Depression and Anhedonia

**Figure 2:**
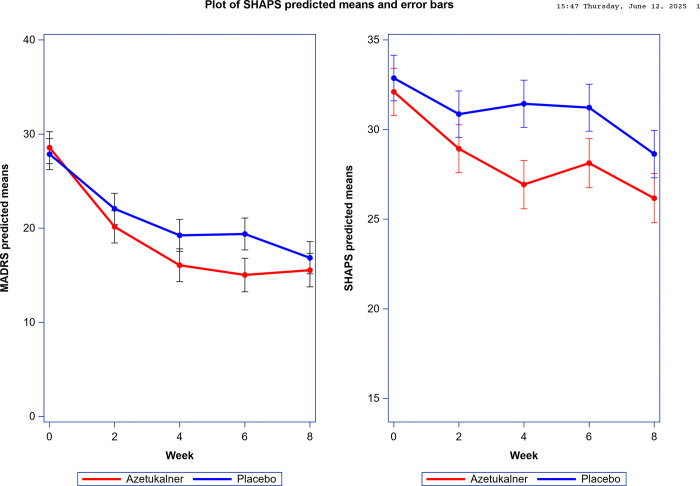
Change in MADRS and SHAPS Score over the 8-Week Treatment Period A. Predicted mean Montgomery-Asberg Depression Rating Scale (MADRS) scores at each study visit. B. Predicted mean Snaith-Hamilton Pleasure Scale (SHAPS) scores at each study visit. The predicted means for were obtained separately for each outcome using a linear mixed-effects model with a random intercept that included treatment, week, and treatment-by-week interaction using all available observations. The bars represent 1 standard error. Scores on the MADRS range from 0 to 60 with higher scores indicating greater severity of depression. Scores on the SHAPS range from 14 to 56 with higher scores indicating greater level of anhedonia.

Changes in MADRS scores did not differ significantly between groups (F 4, 210 = 1.59, p = 0.18). Participants in the azetukalner group showed numerically greater improvement than placebo (Fig. 2, **Table S3**). The largest least squares mean difference between groups was observed at week 6 (difference, −4.35 points; 90% CI, −8.40 to −0.30), favoring azetukalner. Analysis of SHAPS scores did not shows a statistically significant treatment-by-week interaction (F4, 210 = 1.37, p = 0.25). Like MADRS, the azetukalner group showed greater changes in SHAPS scores than placebo (Fig. 2, **Table S3**). The largest least squares mean difference between groups was observed at week 4 (difference, −4.50 points; 90% CI, −7.62 to − 1.39), favoring azetukalner. Between-group differences on the MADRS and SHAPS scales were small at week 8.

### Exploratory Outcomes – Symptom Changes

**Figure 3:**
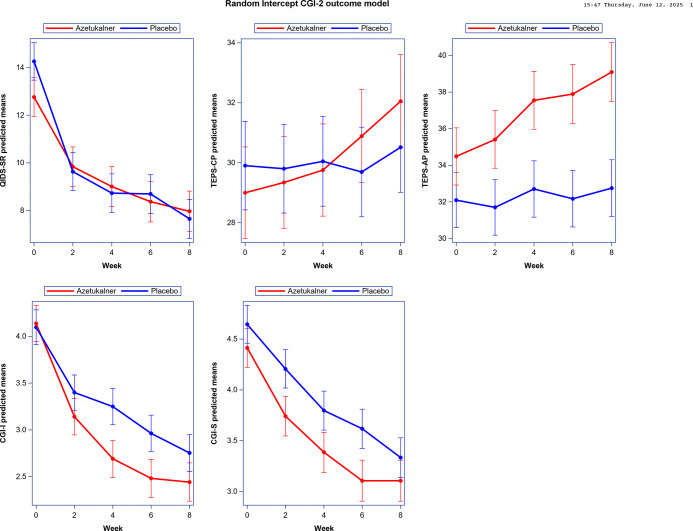
Change in Exploratory Clinical Endpoints over the 8-Week Treatment Period Predicted mean scores of the exploratory endpoints at each study visit. A. Quick Inventory of Depressive Symptomatology-Self Report (QIDS-SR); B. Temporal Experience of Pleasure Scale – Consummatory Pleasure (TEPS-CP); C. Temporal Experience of Pleasure Scale - Anticipatory Pleasure (TEPS-AP); D. Clinical Global Impressions - improvement scale (CGI-I); E. Clinical Global Impressions - severity scale (CGI-S). Predicted means were each separately obtained from a linear mixed-effects model with a random intercept that included treatment, week, and treatment-by-week interaction using all available observations. The bars represent 1 standard error. Scores on the QIDS-SR range from 0 to 27 with higher scores indicating greater severity of depression. Scores on the TEPS-AP range from 10 to 60 and scores on the TEPS-CP range from 8 to 48 with higher scores indicating greater level of pleasure (less anhedonia) on both scales. Scores on the CGI-S range from 1 (Normal; not at all ill) to 7 (Among the most extremely ill patients). Scores on the CGI-I range from 1 (Very much improved) to 7 (Very much worse).

The trajectory of QIDS-SR scores over time was similar between treatment groups (Fig. 3, **Table S5**). Analysis of anticipatory (TEPS-AP) and consummatory (TEPS-CP) pleasure revealed differential effects. Participants in the azetukalner group demonstrated greater improvements in anticipatory pleasure at weeks 2 through 8 (Fig. 3, **Table S5**), with the largest least squares mean difference occurring at week 8 (difference, 6.35; 90% CI, 2.66 to 10.03). No similar pattern emerged for consummatory pleasure at any time point. Participants receiving azetukalner showed larger reductions in illness severity and clinical improvement on clinician-rated CGI-S and CGI-I, respectively, compared to placebo (Fig. 3, **Table S5**). Across study visits, a higher proportion of participants in the azetukalner group were rated as “much” or “very much” improved (CGI-I score ≤ 2) compared to placebo (72.4% [21/29] vs. 54.8% [17/31]). Similarly, 34.5% of the azetukalner group (10/29) were rated as “borderline” or “not at all” ill (CGI-S score ≤ 2) compared to 25.8% (8/31) in the placebo group.

At week 8, the response rate was 37.9% (11/29) in the azetukalner group compared to 35.5% (11/31) in the placebo group (**Table S4**). Remission rates were 31.0% (9/29) in the azetukalner group and 25.8% (8/31) in the placebo group. Eight participants (4 per group) were missing MADRS scores at week 8 and counted as failures on both measures.

### Safety and Tolerability

Over the follow-up period, 27 of 29 participants (93.1%) in the azetukalner group and 25 of 30 participants (83.3%) in the placebo group experienced an AE (Table 2 and **Table S1**). AEs were primarily rated as mild or moderate in severity. In the azetukalner group, the most common AEs were dizziness (11 participants [37.9%]), incoordination (6 [20.7%]), and confusion (6 [20.7%]). In the placebo group, the most common AE was headache (6 [20%]). Urinary-related disorders were the most frequently occurring AESI (6 [20.7%] in azetukalner and 3 [10%] in placebo); all were mild with no need for catheterization or other intervention. Visual hallucinations occurred in 4 participants (13.8%) in the azetukalner group; all but 1 were mild in severity and all resolved. Three participants (10.3%) in the azetukalner group experienced thinking or perceptual disturbances, which led to treatment discontinuation in 1 case. In total, 3 participants in the azetukalner group and 2 in the placebo group discontinued study drug related to AEs. One SAE of fainting was reported in the azetukalner group during the post-treatment phase, occurring 45 days after the last dose of study medication, and was determined to be unrelated to the study drug by the site investigator. No deaths occurred during the trial.

## DISCUSSION

We report the results of a phase II, randomized, parallel-arm, placebo-controlled clinical trial in 60 individuals with MDD who were randomized 1:1 to receive 20 mg of azetukalner or placebo daily with food for an 8-week treatment period. The primary neuroimaging endpoint of the study, change in activation within the bilateral VS during the IFT task from baseline to week 8 post-randomization, was not met. Individuals treated with azetukalner compared to placebo showed benefit on MADRS and SHAPS, although this did not reach statistical significance. Azetukalner showed additional benefit over placebo on a range of exploratory outcomes, including the anticipatory pleasure scale of the TEPS, as well as on CGI-I and CGI-S. Azetukalner was generally well-tolerated, with similar discontinuation rates between drug and placebo.

We previously reported on the brain imaging and clinical outcomes of the first-generation Kv7 opener ezogabine in adults with MDD (15). In that study, we found a non-significant trend towards increased VS activation in response to reward following ezogabine compared to placebo. Our current study of azetukalner showed no trend for difference between the treatment groups in VS activation. Differences in the drug properties and study design may have led to observed differences between outcomes in the current study and the prior one. For one, azetukalner is thought to have a higher specificity for Kv7.2/3 compared to ezogabine (23).

There has been great interest within academia and industry to develop and deploy valid and reliable measures of target engagement in psychiatric clinical trials (24, 25). Indeed, our prior trial and the current were developed within the Experimental Medicine program of the National Institutes of Mental Health (NIMH) with a mandate to deploy estimates of target engagement within experimental medicine projects of novel targets for psychiatric disorders. Our approach utilizing fMRI with reward activation was based in part on the approach utilized within the NIMH Fast Mood and Anxiety Disorders Program (Fast-MAS) wherein fMRI with a reward task similar to the IFT demonstrated evidence of target engagement of a kappa opioid receptor (KOR) antagonist (26, 27). Despite the promise of fMRI to establish target engagement in clinical trials, substantial limitations in terms of validity, replicability and utility of these approach remain.

Our current study did not demonstrate statistical superiority of azetukalner over placebo on the MADRS or SHAPS, which constituted the two secondary outcomes. However, the drug was better than placebo at each week during the dosing period. The difference on MADRS score was particularly large at week 6 where there was a 4.35-point advantage for azetukalner compared to placebo. On the SHAPS, the largest difference was observed at week 4 where there was a 4.5-point advantage of drug compared to placebo. Exploratory outcomes from the TEPS-AP, CGI-I, and CGI-S showed consistent benefit of azetukalner over placebo. Notably, our study was powered to detect an anticipated effect magnitude regarding VS response but was not powered on clinical endpoints. Therefore, our sample size may have been too small to detect a true effect of treatment on our clinical outcomes. Azetukalner 10 mg and 20 mg daily compared to placebo was recently tested as monotherapy in a 6-week, phase II industry-sponsored clinical trial conducted in N = 168 adults with MDD (28). In that study, azetukalner 20 mg daily showed approximately a 3-point advantage over placebo at week 6 on the MADRS scale that did not meet statistical significance. However, azetukalner 20 mg daily compared to placebo did achieve statistical significance on the Hamilton Depression Rating Scale, as well as on the SHAPS. Altogether, the pattern and magnitude of the drug response appear similar across the two studies.

Regarding safety and tolerability, we observed a pattern of AEs that is consistent with prior work concerning azetukalner and other medicines that demonstrate efficacy in seizure disorder. The most commonly observed AEs in the azetukalner group were dizziness, incoordination, and confusion and discontinuation due to AEs were similar between the azetukalner and placebo groups (3 and 2 individuals, respectively).

Limitations of the current study include a small sample size, which could lead to false negative results. The study enrolled at only two sites, which may limit the generalizability of the findings. Another limitation is the sensitivity of the fMRI method used to detect clinically relevant brain effects of the study drug. Our study also included only a 20 mg once daily dose of the study drug. Therefore, we are unable to assess possible dose-response relationships.

In the current study we found that azetukalner compared to placebo had no effect on brain response to reward in individuals with MDD as measured by fMRI. There was lack of statistical separation on the secondary clinical outcomes, although the overall pattern of benefit of azetukalner compared to placebo was consistent with hypothesized drug activity in depression and anhedonia. Phase 3 programs to assess the efficacy and safety of azetukalner in MDD and in bipolar depression are underway and will help inform future research to further characterize the brain effects and potential clinical benefit of drugs targeting the Kv7 channel as antidepressant therapies.

## Supplementary Material

Supplementary Files

This is a list of supplementary files associated with this preprint. Click to download.

• CONSORT2025NPPFremontetalSubmission820.docx

• FremontetalSupplementTP26AUG2025.docx

## Figures and Tables

**Table 1:** Baseline Characteristics of Study Participants

	Azetukalner (N = 29)		Placebo (N = 31)	
	N/ Mean/ Med	%/ SD/ IQR	N/ Mean/ Med	%/ SD/ IQR
Age, years (mean, SD)	39.7	13.2	36.5	12.3
Female sex (N, %)	19	65.5	21	67.7
Race (N, %)^b^				
American Indian or Alaska Native	0	0.0	1	3.2
Asian	5/26	19.2	4	12.9
Black or African American	5/26	19.2	7	22.6
White	14/26	53.8	14	45.2
More than one race	2/26	7.7	5	16.1
Hispanic or Latino (N, %)^b^	7	24.1	8	25.8
At least some college (N, %)	29	100.0	25	80.6
Single, never married (N, %)	15	51.7	16	51.6
Depression Characteristics				
Age at onset of first episode of major depression, years (mean, SD)^c^	22.2	12.7	20.9	12.1
Duration of current episode of major depression, months (median, IQR)^c^	12	6–55	17	4–37
Recurrent episodes of major depression (N, %)	19/23	82.6	20/27	74.1
Depression and Anhedonia Severity				
MADRS score (mean, SD)^d^	28.6	7.0	27.9	6.6
SHAPS score (mean, SD)^e^	32.1	7.9	32.9	6.2
Comorbid Psychiatric Diagnoses				
Anxiety disorder (N, %)	15	51.7	14	45.2
PTSD (N, %)	2	6.9	2	6.5

a IQR denotes interquartile range, MADRS Montgomery-Åsberg Depression Rating Scale, Med median, PTSD post-traumatic stress disorder, SHAPS Snaith-Hamilton Pleasure Scale, SD standard deviation.

b Race, ethnic group and sex were self-reported.

c Age of MDD onset was missing for 1 participant in the azetukalner group. Duration of current MDD episode was missing for 6 participants the azetukalner and 5 in the placebo group.

d Scores on MADRS range from 0 to 60, with higher scores indicating greater depression.

e Scores on the SHAPS range from 14 to 56, with higher scores indicating higher levels of anhedonia.

**Table 2:** Adverse Events by 12 Weeks

	Azetukalner (N = 29)		Placebo (N = 30)	
	N	%	N	%
Any adverse event^a^	27	93.1	25	83.3
Moderate or severe adverse event	18	62.1	12	40.0
Serious adverse event	1	3.4	0	0.0
Adverse event related to study treatment^b^	24	82.8	15	50.0
Most common adverse events^c^				
Dizziness	11	37.9	3	10.0
Incoordination	6	20.7	1	3.3
Confusion	6	20.7	0	0.0
Drowsiness	4	13.8	4	13.3
Fatigue	4	13.8	3	10.0
Visual hallucinations	4	13.8	0	0.0
Headache	3	10.3	6	20.0
Blurred vision	3	10.3	0	0.0
Brain fog	3	10.3	0	0.0
Eye floaters	3	10.3	0	0.0
Impairment of attention	3	10.3	0	0.0
Memory impaired	3	10.3	0	0.0
Word finding difficulty	3	10.3	0	0.0
Nausea	2	6.9	3	10.0
Back pain	1	3.4	3	10.0
Upper respiratory tract infection	1	3.4	3	10.0
Adverse events of special interest^d^				
Any adverse event of special interest	12	41.4	3	10.0
Urinary-related^e^	6	20.7	3	10.0
Visual hallucinations	4	13.8	0	0.0
Thinking disturbances	2	6.9	0	0.0
Perceptual disturbance	1	3.4	0	0.0
Tachycardia	1	3.4	0	0.0
Visual phenomena	1	3.4	0	0.0

a Data for adverse events from randomization through 12 weeks are shown. Adverse events were collected using MedDRA-preferred terms. Safety was evaluated in the safety population and included all randomized participants who received at least one dose of azetukalner or placebo.

b Adverse events determined by the investigator to be possibly, probably, or definitely related to azetukalner or placebo were considered treatment-related.

c The most common adverse events were events experienced by at least 10% of participants in either the azetukalner or placebo group.

d Adverse events of special interest included events suggestive of disturbances in thinking and perception, events suggestive of urinary retention, events suggestive of cardiovascular changes, events suggestive of ophthalmologic changes, and clinically significant ECG results.

e Urinary-related adverse events included urinary frequency, urinary hesitation, urinary retention, urinary urgency, urinary straining, and weak urinary stream.
